# 100 years post-insulin: immunotherapy as the next frontier in type 1 diabetes

**DOI:** 10.1093/immadv/ltab024

**Published:** 2021-11-24

**Authors:** James A Pearson, Eoin F McKinney, Lucy S K Walker

**Affiliations:** 1 Diabetes Research Group, Division of Infection and Immunity, School of Medicine, Cardiff University, Cardiff, Wales, UK; 2 Cambridge Institute of Therapeutic Immunology and Infectious Disease, Jeffrey Cheah Biomedical Centre, Cambridge, England, UK; 3 Department of Medicine, University of Cambridge School of Clinical Medicine, Cambridge, England, UK; 4 Cambridge Centre for Artificial Intelligence in Medicine, University of Cambridge, Cambridge, England, UK; 5 Division of Infection and Immunity, Institute or Immunity and Transplantation, University College London, Royal Free Campus, London, UK

**Keywords:** type 1 diabetes, human, NOD mouse, immunotherapy

## Abstract

Type 1 diabetes (T1D) is an autoimmune disease characterised by T cell-mediated destruction of the insulin-producing β cells in the pancreas. Similar to other autoimmune diseases, the incidence of T1D is increasing globally. The discovery of insulin 100 years ago dramatically changed the outlook for people with T1D, preventing this from being a fatal condition. As we celebrate the centenary of this milestone, therapeutic options for T1D are once more at a turning point. Years of effort directed at developing immunotherapies are finally starting to pay off, with signs of progress in new onset and even preventative settings. Here, we review a selection of immunotherapies that have shown promise in preserving β cell function and highlight future considerations for immunotherapy in the T1D setting.

## Introduction

T1D is a complex T cell-mediated autoimmune disease, resulting in destruction of the insulin-producing β cells and a deficiency in insulin secretion. Prior to the discovery of insulin in 1921, individuals with T1D would have died within a year or two of diagnosis [1]; however, since the discovery and mass production of insulin, T1D is no longer a death sentence and the condition can be managed by exogenous insulin, either delivered by multiple daily injections or a pump. Nevertheless, over time many patients develop complications including cardiovascular disease, retinopathy, neuropathy, and nephropathy.

**Figure 1:**
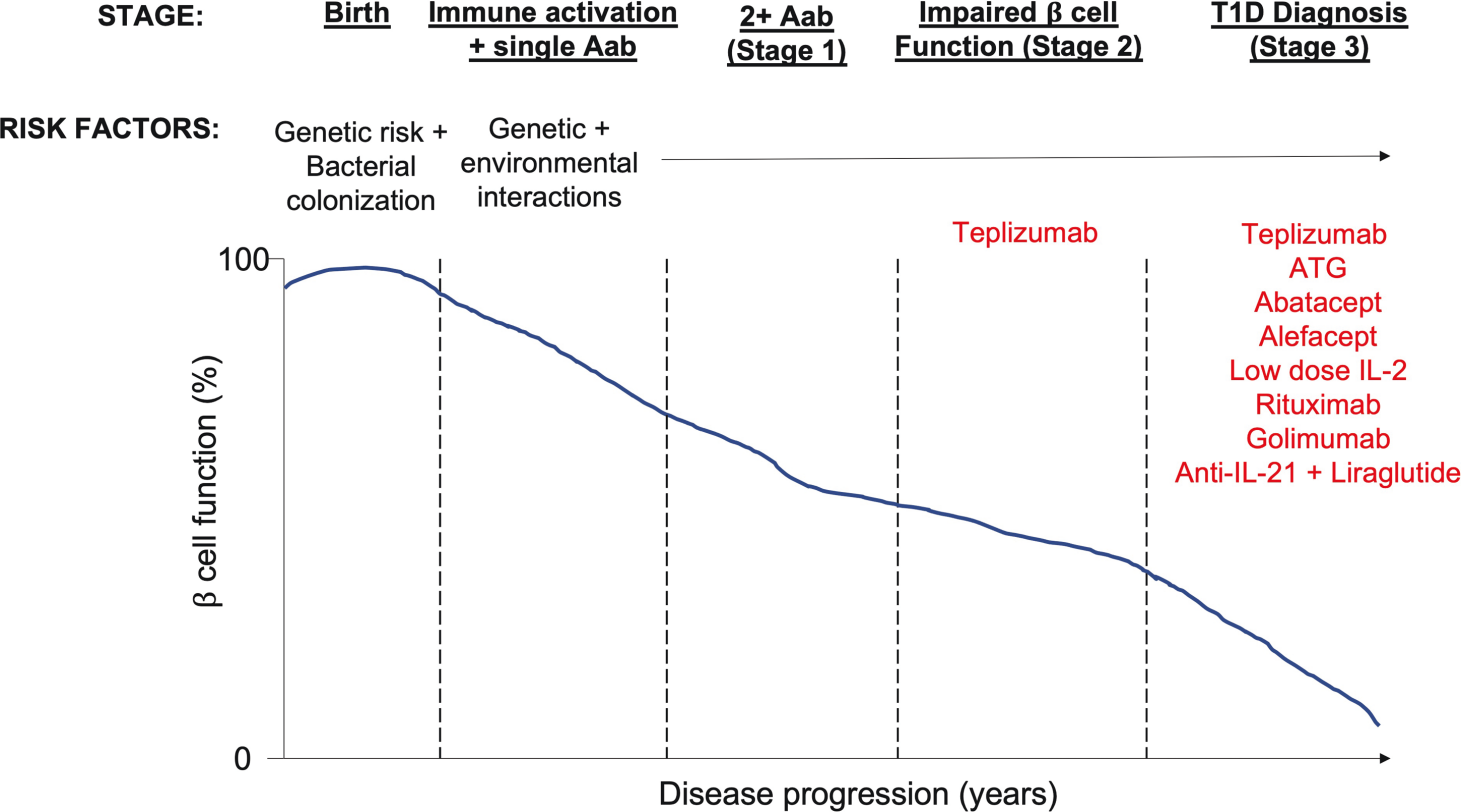
Stages of T1D development and immune interventions. From birth individuals inherit a genetic predisposition to developing T1D, as well as a collection of colonising bacteria. In those individuals with a risk of developing T1D, the immune interactions with environmental modifiers can lead to inappropriate activation of the immune system driving autoreactive T and B cells and the secretion of detectable autoantibodies (stage 1). The immune response impairs the function and survival of the insulin-producing islet β cells, resulting in a dysglycemic state (stage 2) and finally the clinical diagnosis of T1D when a sufficient number of beta cells have been destroyed (stage 3). Immunotherapy studies have largely been conducted in those with recent-onset T1D, with the exception of Teplizumab, which has also been conducted in ‘at risk’ individuals. Key trials discussed in this article are highlighted in the figure.

**Figure 2:**
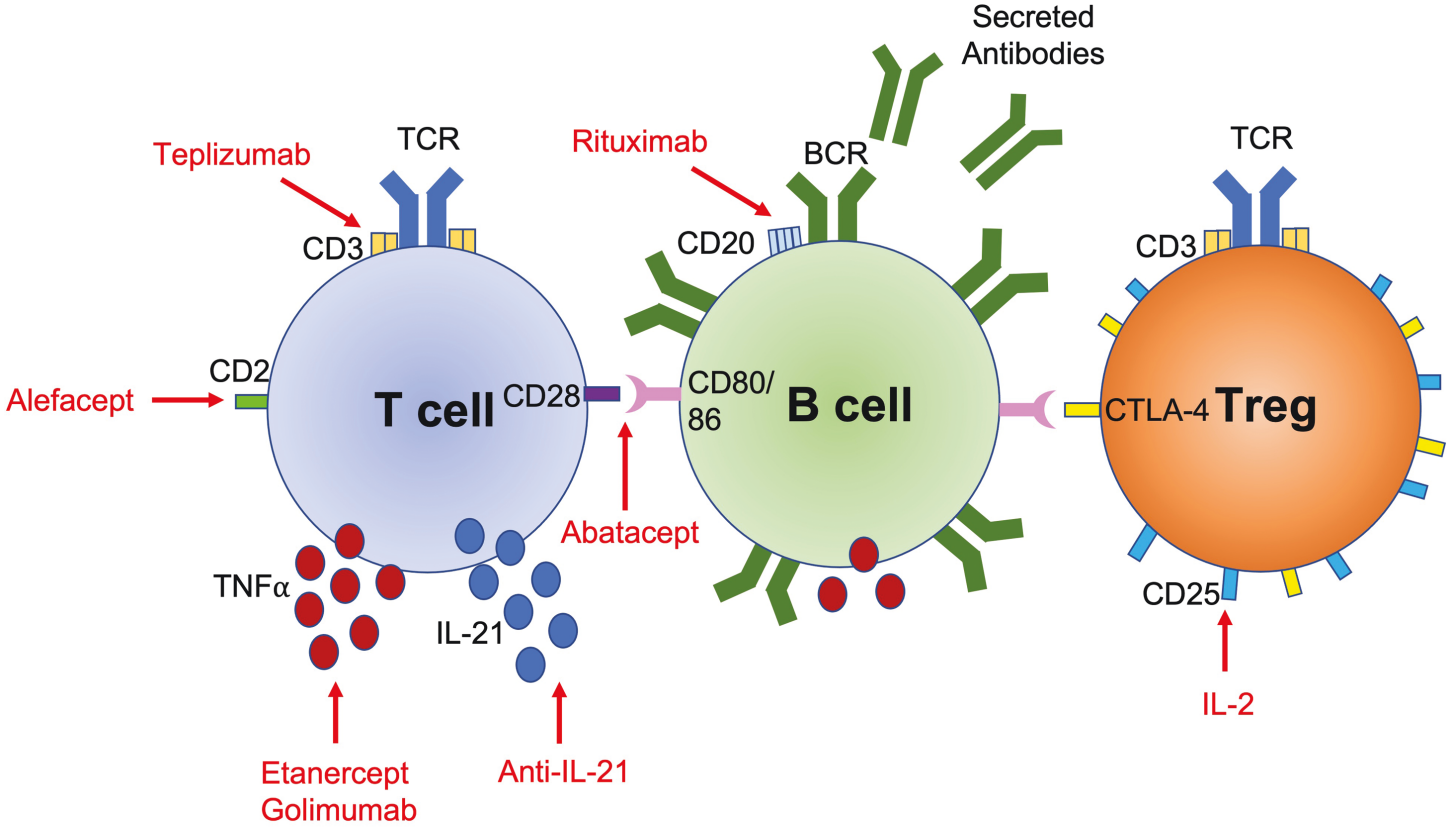
Immune intervention targets. Schematic illustrating key immunotherapies that have been tested in the T1D setting and their immune cell targets. Many immunotherapies target markers expressed by T cells, others target B cells or cytokines. Immunotherapies can also target regulatory T cells (Tregs) e.g. IL-2, which helps to expand and boost Treg suppression, preventing the destruction of the islet β cells. Immunotherapies are in red with arrows indicating their targets. While a B cell is shown interacting with T cell CD28, this costimulation signal can come from CD80/86 on other immune cells such as macrophages and dendritic cells.

Clinical diagnosis of T1D occurs relatively late in the disease process when a large number of β cells have been destroyed by islet autoantigen-specific T cells; however, there are several preclinical stages in which the immune response has already been triggered and is actively responding to pancreatic islet antigens (Fig. 1; [2]). This presents a window of opportunity to potentially intervene and reset the immune system prior to extensive tissue damage. Autoantibodies, secreted by B cells, can be detected against a number of islet antigens, with multiple autoantibody specificities associated with an increased risk of progression to T1D diagnosis [3–7]. These autoantibodies, alongside genetic susceptibility, provide a crucial biomarker pre-diagnosis to identify those at most risk and who may be the best candidates for future immunotherapy aimed at delaying or preventing T1D development [8].

Based on successful experiments in the Bio-breeding rat model of type 1 diabetes [9], early attempts at immunomodulation included the use of the calcineurin inhibitor cyclosporin [10]. Of 30 patients treated within 6 weeks of diagnosis, 16 reverted to having normal C-peptide levels and became insulin-independent, an unprecedented result. The use of corticosteroids plus daily azathioprine also showed beneficial outcomes in new onset T1D, with 50% of the treatment group showing C-peptide levels >0.5 nmol/l (three being insulin-independent) compared to 15% of the control group (none being insulin-independent) [11]. Although these approaches were not pursued due to problematic side effects, the trials were nevertheless important in demonstrating the potential of immunomodulation in T1D. There have since been multiple immunotherapy studies aimed at curtailing the loss of β cells by targeting the key immune cells involved in the disease process, as well as cytokines that they produce (Fig. 2). In addition, therapies which may boost immune regulation have also been studied. Here, we review the key successful non-antigen-specific immunotherapies that show most promise in preserving β cell function or even delaying T1D development. Antigen-specific approaches are not covered in this article and have been recently reviewed elsewhere [12].

## T cells in T1D

CD4^+^ and CD8^+^ T cells orchestrate the inflammatory process that culminates in the destruction of the islet β cells, leading to the development of T1D. Many of the genes associated with susceptibility to type 1 diabetes are active in T cells and the strongest genetic contribution to disease maps to the human leukocyte antigen genes, which function to present antigens to T cells. T cells infiltrate the pancreatic islets in people with diabetes [13, 14] and in mouse models, diabetes can be transferred from one animal to another by the adoptive transfer of T cells [15]. Thus, extensive effort has been directed at the development of immunotherapies to target T cells.

## Anti-CD3 immunotherapy

In 1979, a mouse hybridoma cell line was developed that produced an IgG2a monoclonal antibody, named Orthoclone (OKT3), against a T cell surface antigen [16], later identified as the *ε* chain of the CD3 receptor [17]. In 1981, the first patients were administered OKT3, which was shown to successfully reverse allograft rejection [18, 19]. In 1985, OKT3 became commercially available for use in transplantation, making it the first-in-human approved monoclonal antibody; however, by the late 1980s, OKT3 use in the clinic became limited following the severe cytokine release from activated T cells [20–22]. This cytokine release was induced by OKT3 crosslinking with the T-cell receptor/CD3 complex; however, binding of the Fc portion of OKT3 by Fc-receptor-expressing cells further enhanced the crosslinking and thus the severity of the cytokine release. Activation of the T cell also varied with the antibody isotype of the OKT3 antibody, with IgG2a having the strongest immunostimulatory effect [23, 24]. As OKT3 was a mouse anti-human antibody, human anti-mouse antibodies were also raised against OKT3, which resulted in clearance of OKT3 and a reduction in efficacy [20]. Thus, to improve clinical efficacy and tolerance, OKT3 antibodies were humanised and developed with modified Fc portions to prevent Fc binding by Fc receptors and thus severe cytokine release, while preserving their suppressive effects. Teplizumab is a modified OKT3 antibody, with the same binding region as OKT3 but the amino acids at positions 234 and 235 of the human IgG1 were substituted with alanine (hOKT3 γ1(Ala-Ala)) [25].

Pivotal pre-clinical studies by Lucienne Chatenoud and colleagues showed short-term anti-CD3 treatment (5-day course) was able to induce disease remission in up to 80% of recently diagnosed diabetic non-obese diabetic (NOD) mice, and this was associated with a transient and partial T cell depletion, with numbers returning to normal within 15–20 days [26, 27]. This protection was not due to deletion of autoreactive T cells, as insulitis was only transiently reduced, and spleen cells from these mice could transfer diabetes to irradiated mice [27]. The protective effect of anti-CD3 treatment in mice may relate to the induction of regulatory T cells (Treg) and immunosuppressive cytokines (TGFβ) [28–30] and, partial TCR signalling leading to the clonal anergy or age-dependent deletion of specific T cells [31, 32]. Transgenic NOD mice were also developed to express human CD3 [33], providing a useful preclinical model for testing humanised anti-CD3 antibodies. Following anti-CD3 treatment, diabetes in these mice was reversed and again, in line with previous data [29], protection was TGFβ-dependent and associated with enhanced Treg function [33].

Given the success of the pre-clinical studies of anti-CD3 treatment in NOD mice, Herold and colleagues recruited 24 newly diagnosed individuals with T1D (within 6 weeks of diagnosis), half of whom received an escalating dose of Teplizumab each day for 2 weeks, while the placebo group received no antibody [34]. Importantly, 12 months after treatment, two thirds of the Teplizumab-treated group had C-peptide responses that were equivalent or higher than their response at study entry, whereas 10 out of the 12 control participants exhibited a decline in C-peptide response. Similarly, a phase II study of another humanised anti-CD3 antibody (Otelixizumab) in 80 individuals with new onset T1D also showed a slower deterioration of β cell function in those receiving anti-CD3 treatment [35]. Preservation of even a small amount of residual insulin secretion, measured by C-peptide, can provide long-standing health benefits [36]. Later anti-CD3 studies confirmed this preservation of insulin secretion by the β cells could be maintained for many years [37, 38], with the latest data indicating up to 7 years post-diagnosis [39]. A phase III trial of Teplizumab in 516 individuals however failed to meet its primary endpoint, a composite outcome comprising insulin dose and haemoglobin A1c (HbA1c) which had not been previously validated [40]. However, exploratory analyses showed that C-peptide declined less in the treated group than in the placebo group and that 5% of patients were not taking insulin at 1 year compared with no patients in the placebo group.

As mentioned above, the fact that islet autoantibodies are produced many years prior to diabetes development provides a window of opportunity for therapeutic intervention. Herold and colleagues set out to exploit this window by administering Teplizumab to high-risk relatives of patients with T1D who had dysglycemia and the presence of 2 or more islet autoantibodies but had not yet been diagnosed with T1D [41]. This study successfully delayed the development of T1D in these individuals, with 57% of the teplizumab group being diabetes free compared to 28% of the placebo group. An extended follow-up study (median of 923 days) found that 50% of the Teplizumab-treated group were still diabetes free compared to 22% of the placebo group [42]. These data have changed the landscape for immunotherapy in T1D, providing the first evidence that T cell-directed therapies administered in at risk individuals can alter the future disease course.

This prevention of β cell destruction has been associated with a combination of induced regulatory T cell responses [43], partially exhausted CD8^+^ T cells, characterised by TIGIT and killer cell lectin-like receptor G1 expression, which were associated with improved clinical efficacy [42, 44], and reduced proinflammatory cytokines [42]. The success of this intervention in delaying T1D development is groundbreaking and Teplizumab is likely to be the first immunotherapy licensed for delaying, and possibly preventing, the development of T1D. There is still work to be done: trials in T1D present significant challenges in the areas of recruitment and endpoints so accumulating sufficient data is problematic and the US Food and Drug Administration rejected a request for Teplizumab approval in July 2021; however, momentum is clearly building for immunotherapies to be approved in the T1D setting and Teplizumab looks to be at the forefront. Further longitudinal studies are needed to identify the duration for which T1D can be delayed following a single course of additional anti-CD3 treatment and whether additional doses, or other combination treatments, could be administered later to maximise clinical efficacy.

## Anti-thymocyte globulin (ATG)

ATG is a polyclonal IgG targeting multiple T cell antigens and mediating cellular depletion: in NOD mice, similar to anti-CD3 administration, ATG treatment was able to reverse diabetes in mice with recent-onset disease [45]. Initial small studies in humans with recent-onset T1D suggested ATG administration may help preserve β cell function [46, 47]; however, in a phase II randomised multi-center, placebo-controlled trial involving 58 individuals within 100 days of T1D diagnosis, ATG (6.5 mg/kg administered over a 4-day course) did not preserve β cell function [48, 49]. This failure was linked to a decrease in the Treg to T-effector memory ratio in ATG treated individuals between baseline and 6 months, since effector memory CD4^+^ T cells were poorly depleted relative to the other T cell subsets examined [48]. A subsequent trial in 25 individuals with established T1D (between 4 months and 2 years post-diagnosis) used a lower dose of ATG (2.5 mg/kg administered as 0.5 mg/kg on day 1 and 2 mg/kg on day 2) and combined the treatment with Granulocyte colony-stimulating factor (GCSF) in line with previous preclinical data [45]. This approach appeared to result in protection of the β cells; on average, subjects who received placebo experienced a 39% reduction in C-peptide over 1 year, while those who received ATG/GCSF experienced a 4.3% increase over the same time period [50]. The relative resistance of CD4^+^ effector memory T cells to depletion was also evident in this trial, but Tregs appeared to be preserved [51, 52]. A further trial in 89 individuals within 100 days of T1D diagnosis confirmed that low-dose ATG (2.5 mg/kg) resulted in clinical benefit, with C-peptide levels 57% higher in recipients of low-dose ATG, compared with recipients of placebo, at the 1-year timepoint [53]. Side-by-side comparison with the ATG/GCSF combination revealed that the low-dose ATG monotherapy was favourable [53], and a separate study confirmed that GCSF alone did not preserve β cell function [54]. Thus, low-dose ATG remains an interesting candidate for further development. Notably, attempts to compare β cell preserving interventions across multiple clinical trials identified low-dose ATG and anti-CD3 immunotherapy as the treatments showing the greatest impact on C-peptide preservation [55].

## Abatacept

A further example of a T cell-directed immunotherapy is the cytotoxic T lymphocyte-associated antigen 4 (CTLA-4)-Ig fusion protein, Abatacept. CTLA-4 is naturally expressed at high levels in regulatory T cells; it interacts with the same ligands as the T cell costimulatory receptor CD28 but binds to them with higher affinity. CTLA-4-Ig fusion proteins such as Abatacept therefore bind to the costimulatory ligands CD80 and CD86 on antigen-presenting cells and inhibit their interaction with T cell CD28 ([56]; Fig. 2). Since CD28 costimulation provides an important ‘second signal’ to promote full T cell activation, inhibiting this with CTLA4-Ig would be expected to be immunosuppressive. When CTLA-4-Ig was administered to NOD mice early, around the time insulitis first develops, only 11% of the mice went on to become diabetic compared with 87% in the control-treated group, while later administration had little effect [57]. Surprisingly, however, mice expressing CTLA-4-Ig transgenically from birth showed exacerbated diabetes development, with 100% of mice diabetic by 24 weeks compared with only 8.3% of the non-transgenic NOD mice [58]. This is now thought to reflect the role of CD28 in the development of Tregs which have immunosuppressive function [59]. In the context of Treg deficiency, and an absence of CD28 signalling since birth, it was proposed that alternative costimulatory pathways had compensated for the lack of CD28 in these mice [58]. Therapeutic targeting of the CD28 pathway therefore requires careful consideration regarding impacts on the Treg population, and timing of administration is likely to be important.

The CTLA-4-Ig molecule Abatacept was trialled in 112 patients (6–45 years of age) diagnosed with T1D within the last 100 days. Infusions were given intravenously on days 1, 14, and 28 and then monthly for 2 years. Abatacept was able to slow the decline in β cell destruction and function for an estimated 9.6 months [60], with higher C-peptide levels still observed in the treated group, compared to placebo, 1 year after therapy cessation [61]. This protective effect was associated with a reduced CD4^+^ central memory T cell population and B cell population, as well as increased Tregs and naive CD4^+^ T cells [62]. Recent data suggest that analysis of circulating follicular helper T cells (Tfh) prior to Abatacept treatment may prove useful in predicting the subsequent clinical response [63]. Following on from the study in new onset T1D, Abatacept is currently being trialled in people at risk of T1D development (NCT01773707).

### Alefacept

Alefacept is another Ig fusion protein comprising two LFA-3 molecules bound to the Fc portion of human IgG1. It binds to CD2 and mediates depletion of antigen experienced effector/memory T cells. Memory T cells are an attractive target in autoimmune disease and are believed to be less sensitive to costimulation blockade drugs such as Abatacept. Alefacept inhibits the proliferation of T cells in mixed lymphocyte reactions in a manner that depends on Fc-receptor binding [64]. In the T1DAL study, Alefacept was delivered by intramuscular injection in two 12-week courses to 33 individuals within 100 days of T1D diagnosis, while 16 individuals received placebo treatment [65]. The primary endpoint was not met, since the difference between C-peptide measurements in a 2h mixed-meal tolerance test (MMTT) at 12 months was not significant (*P* = 0.065); however, the secondary endpoint, involving C-peptide measurement in a 4-h MMTT at the same timepoint was met (*P* = 0.019). It was suggested that the curtailed recruitment following voluntary withdrawal of Alefacept by the manufacturer may have reduced the power to detect the impact of treatment. Follow-up analysis suggested beneficial effects were maintained 15 months after therapy cessation, with Alefacept-treated individuals exhibiting higher C-peptide levels than placebo-treated individuals, a significantly lower insulin requirement, and substantially lower rates of hypoglycaemia [66].

### IL-2 therapy

An additional immunotherapeutic approach directed at T cells centres on the selective expansion of Tregs using the cytokine IL-2. Although IL-2 was originally described as a growth factor for conventional T cells, it subsequently became clear that a major biological function for IL-2 is to regulate immune responses by supporting the homeostasis of Tregs. A role for IL-2 in immune regulation indicated why defects in IL-2, or in genes that contribute to IL-2 signalling, are associated with autoimmune diseases, including T1D [67, 68]. Since Tregs express high levels of CD25, a component of the high-affinity IL-2 receptor, this sparked the idea that they might be selectively targeted by low doses of IL-2. The use of IL-2 to suppress immune responses is an extraordinary example of the same agent being used at different doses for opposing purposes, since high-dose IL-2 is used to promote anti-tumour responses in cancer patients.

In preclinical models, low-dose IL-2 was able to expand Tregs and reverse established type 1 diabetes [69]. However, a human phase I study in which IL-2 was combined with rapamycin gave disappointing results: nine individuals within 4 years of T1D diagnosis were included and although Treg frequencies increased, C-peptide transiently decreased and this coincided with an increase in NK cells and eosinophils [70]. In the light of this, the field has moved in two directions: one involving careful dosing to identify regimens that activate Tregs without activating effector cells [71], and the other involving the generation of mutant IL-2 therapeutics aimed at avoiding the detrimental activation of NK cells, eosinophils, and effector T cells. Encouragingly, doses of IL-2 that can be safely administered have now been identified and can be further explored in larger patient groups [72]. At the same time, numerous IL-2 mutant approaches are in development [73–75]. Boosting Treg numbers by cell therapy in combination with IL-2 administration is also being explored, however, the data again reinforce the need for IL-2 mutant approaches since NK cells, mucosal-associated invariant T cells and CD8^+^ T cells were also affected by IL-2 administration [76]. While we do not cover cell therapy in this review, it should be noted that Treg cell therapy is an area of emerging interest as has been discussed elsewhere [77, 78].

## B cells in T1D

B cells are also implicated in the development of T1D. In animal models, B cell deficiency [79] or B cell depletion [80] inhibits the onset of diabetes and to our knowledge, only one individual lacking B cells, caused by X-linked agammaglobulinemia, has developed T1D [81]. Autoantibodies, secreted by B cells, can be detected against a number of islet antigens including insulin, glutamic acid decarboxylase, tyrosine phosphatase-related islet antigen 2 (IA-2) and zinc transporter 8 (ZnT8) [3–6]; however, autoantibodies themselves are not believed to be pathogenic. Importantly, there is a substantially increased risk in those individuals who have 2 or more islet antigen-specific autoantibodies, with an 84% risk of developing T1D by 18 years of age [7]. Thus, the presence of autoantibodies provides an important biomarker pre-diagnosis, when individuals are still normoglycemic, that correlates with disease progression risk. B cells are also potent antigen-presenting cells, capable of activating autoantigen-specific T cells to cause diabetes [79]. B cells have been shown to infiltrate the islets, with increased islet CD20^+^ B cell presence associated with enhanced β cell destruction, and diagnosis of T1D at an earlier age, compared to those with fewer CD20^+^ islet B cells [13, 50, 82]. Thus, B cells have also been targeted in immunotherapy trials in T1D.

### Anti-CD20 immunotherapy

Rituximab binds to CD20 expressed on the surface of B cells, leading to their destruction mediated via antibody-dependent cell-mediated cytotoxicity, apoptosis, and complement-dependent cytotoxicity [83, 84]. It is important to note that Rituximab does not deplete all B cells, as plasma cells, which secrete antibodies, do not express CD20 and other B cell subsets such as B1 cells, germinal centre B cells and tissue-resident B cells may be less sensitive to depletion. Rituximab has shown efficacy in a number of different autoimmune diseases including systemic lupus erythematosus and rheumatoid arthritis. To explore the benefit of B cell depletion in T1D, pre-clinical studies were conducted by studying transgenic hCD20 NOD mice, expressing human CD20 on B cells [80]. In these hCD20 NOD mice, a single cycle of anti-CD20 antibody administration (9-day treatment cycle: 0.5 mg/ml at day 0, followed by three injections of 0.25 mg/ml at 3-day intervals), was able to both delay and reduce the development of T1D. Importantly, similar to anti-CD3 immunotherapy [26], anti-CD20 administration was able to reverse diabetes, with over one third of mice in remission. Studies evaluating the repopulation of cells in NOD mice found that the protection of anti-CD20 mice was associated with increased regulatory immune cells [80, 85–87] and reduced proinflammatory cytokines secretion by, and activation of, islet T cells [88].

Following the positive outcomes in pre-clinical models, Rituximab was trialled in individuals with recent-onset T1D. The therapy was administered by intravenous infusion in 4 doses over a period of 22 days, with 49 individuals receiving Rituximab and 29 receiving placebo [89]. The primary outcome measure was stimulated C-peptide levels in a MMTT 1 year after the initial infusion. Results from this clinical trial suggested clear benefit of B cell depletion: mean C-peptide levels were 20% higher in the Rituximab-treated group (0.56 pmol/ml in the Rituximab group vs. 0.47 pmol/ml in the placebo group) and treatment also significantly reduced glycated haemoglobin levels (6.76% vs. 7.00%) and decreased the required insulin dose (0.39U ± 0.22/kg of body weight vs. 0.48 U ± 0.23/kg). In common with other clinical trials, the initial improvements were short-lived and C-peptide continued to decline thereafter. Of note, CD19^+^ B cell counts had substantially recovered by 6 months post-treatment initiation suggesting the possibility that benefits might be increased if the depletion could be sustained. In addition, the participants recruited to this study were aged 8–40 years of age [89]; however, given the prevalence of B cells in the pancreas of individuals diagnosed with T1D before 7 years of age [50], it is possible that Rituximab may show enhanced clinical efficacy in younger individuals and possibly in the ‘at risk’ population.

### Targeting inflammatory cytokines in T1D

IL-1β secretion increases with progression to diabetes and islet β cell destruction [90, 91]; however, two randomised, double-blind, placebo-controlled trials administering either Canakinumab (a human anti-IL-1 monoclonal antibody) or Anakinra (a human IL-1 receptor antagonist) were conducted, which failed to show any protective effects. This is in line with data from NOD mouse studies of IL-1 receptor- or IL-1β-deficient NOD mice, where no protection from diabetes development was observed [92, 93]. Likewise, blocking IL-6Ra in a recent trial in individuals with newly diagnosed T1D does not appear to provide benefit [94].

TNFα has been a target of interest in T1D for some time. Studies in NOD mice have yielded complex results, suggesting that TNFα plays site-specific, cell type-specific and age-dependent roles [95]. Administering anti-TNFα antibody to neonatal mice robustly inhibited the development of diabetes and this was associated with decreased T cell responses to islet antigens [96]; however, protection was weaker if treatment was initiated in adult mice, and administering TNFα itself exacerbated disease in neonates but paradoxically delayed it in adults. TNFR1-deficient NOD mice are however protected from the development of T1D [97].

Serum TNF is increased in individuals with recent-onset T1D [98] and TNFα is known to be toxic to the islet β cells [99]. Thus, TNFα-targeting therapies were administered to newly diagnosed T1D patients to test whether they could preserve β cell function. Etanercept, a recombinant soluble TNF-receptor fusion protein that binds to TNFα was trialled in 18 subjects with newly diagnosed T1D. In this randomised, double-blind, placebo-controlled feasibility study over 24 weeks, Etanercept was shown to increase mean C-peptide levels by 39% from baseline whereas a mean decrease of 20% was observed in the placebo group [100]. More recently, Golimumab, an anti-TNFα monoclonal antibody previously approved for the treatment of rheumatoid arthritis and ulcerative colitis, has also been tested in newly diagnosed individuals with T1D [101]. This phase II randomised, double-blind, placebo-controlled study involved 56 participants receiving Golimumab and 28 participants receiving placebo, and resulted in significantly higher C-peptide and lower insulin use in the treatment group after 52 weeks. Since reagents that block the TNFα pathway are widely used in rheumatology settings and approved for use in patients as young as 2 years of age, this requires further investigation in T1D.

Interleukin 21 (IL-21) has also gained some traction as a target for T1D immunotherapy. IL-21 is the characteristic cytokine made by follicular helper T cells (Tfh cells), that provide help for B cell antibody production, and therefore plays an important role in humoral immunity [102]. NOD mice lacking IL-21 or IL-21 receptor were protected from diabetes development, while transgenic expression of IL-21 in pancreatic islets was sufficient to induce diabetes in non-autoimmune prone (C57BL/6) mice [103, 104]. In a TCR transgenic mouse model of diabetes, T cells responding to islet antigen showed a Tfh phenotype with high IL-21 expression, and the pancreas-infiltrating T cells were shown to express IL-21, IFNγ, and TNFα [105]. In humans, a genetic region encompassing the IL-2 and IL-21 genes is associated with T1D [106] and an increased proportion of effector memory CD4^+^ T cells secreting IL-21 and elevated Tfh cells have been reported in people with T1D compared to healthy controls [105, 107]. Interestingly, the gene expression of cells responding to pro-insulin in genetically at risk children showed elements of a Tfh signature (including *IL-21*), with a transition to a Th1-like signature (with decreased *IL-21* and increased *IFNG* and *TNF*) after the appearance of autoantibodies [108]. A phase II randomised double-blind, double-dummy, placebo-controlled study was conducted in recent-onset individuals with T1D, where they received either anti-IL21, anti-IL-21 with liraglutide, liraglutide alone or placebo (77 individuals per treatment arm) [109]. Liraglutide is a glucagon-like peptide 1 receptor agonist, which works by increasing insulin secretion from the pancreas and decreasing glucagon release. Thus, liraglutide improves β cell function. Von Herrath and colleagues found that in all treated groups, HbA1C was lowered compared to placebos; however, in the combination of anti-IL-21 with liraglutide, a smaller reduction in C-peptide following a MMTT was observed, suggesting enhanced β cell function in the combination group, compared to single treatment groups. It will be important to enlarge the study and determine how long the effects may last following cessation of treatment.

### Microbial-derived therapeutics

Environmental factors such as the intestinal bacterial composition are important for shaping the immune response and modulating susceptibility to T1D. Altered bacterial composition has been reported in both individuals with T1D, and those ‘at risk’ of T1D development [110–118]. These changes in bacterial composition have also been linked to the development of early β cell autoantibody responses [114]. Studies in NOD mice have also suggested that antibiotic administration, through depleting components of the bacterial composition, can alter immune responses and susceptibility to T1D development [119–126]. In humans, while antibiotic use alters the gut bacterial composition, particularly in the first few months of life [118], it does not seem to strongly associate with the development of islet autoimmunity or T1D [117, 127], although it can reduce beneficial Bifidobacteria (a probiotic) members [118]. Probiotics, bacteria with potential health benefits, have also been studied for their role in modulating susceptibility to T1D. *L. casei*, VSL#3 (a mixture of *B. longum, B. infantis*, *B. breve*, *L. acidophilus, L.casei, L. delbrueckii subsp. L. bulgaricus*, *L. plantarum*, and *Streptococcus salivarius subsp. Thermophilus*) and IRT5 (a mixture *of L. acidophilus, L. casei, L. reuteri*, *Bifidobacterium bifidum*, and *Streptococcus thermophiles*) have all been shown to protect NOD mice from developing T1D by promoting tolerogenic immune responses and reducing inflammatory Th1 cells [128–131]. *Lactobacillus johnsonii* N6.2, another probiotic, has also been shown to protect bio-breeding diabetes-prone rats from developing T1D [132, 133]. This probiotic has also been used in human studies whereby in a double-blind randomised trial in healthy adults, *L. johnsonii* N6.2 was safe and induced tolerogenic immune responses [134]. Studies are currently ongoing in children, adolescents and adults with T1D to identify safety and tolerance to *L. johnsonii* N6.2, as well as the immunological responses (NCT03961854 and NCT03961347). *Bifidobacterium longum subsp. infantis* is also being evaluated as a probiotic (NCT04769037), due to its ability to metabolise human milk oligosaccharides, which have significant impacts on inducing tolerogenic immune properties [135]. To date, only one human study has shown success in limiting β cell destruction. A faecal microbiota transplant (FMT) study conducted in individuals with recent-onset (<6 weeks) T1D, showed participants receiving autologous FMTs, compared to allogenic (healthy control) FMTs, had improved preservation of β cell function for the 12 months individuals were followed post-FMT [136]. Thus, the area of microbial-derived therapeutics for T1D is still in an early stage of development and more work is required to determine whether it can be harnessed to modulate the immune response and deliver long-term clinical benefits.

## Future directions

In the 100 years since the discovery of insulin, there is still no cure for T1D; however, the promise of immunotherapy is gradually starting to be realised, with early signs of progress in both prevention and new onset settings. Key challenges moving forward lie in discerning which interventions are best suited to which disease stage; intervening after the emergence of symptomatic disease (stage 3; Fig. 1) will likely require memory cell targeting, while preventative interventions (stage 1/2) in children will need to have excellent safety profiles. A better appreciation of disease endotypes, for example, related to age of diagnosis [137], will ultimately inform the stratification of individuals to different treatment options. Using biomarkers, such as Tfh [63], or Treg and soluble IL-2R [72] to unpick the heterogeneity in clinical response will also be key.

Capitalising on the window to prevent T1D development will require extensive screening initiatives to identify at-risk individuals. Studies suggest that 95% of children who progress to clinical diabetes in puberty have autoantibodies by the age of 5; however, the time from seroconversion to clinical disease can vary enormously, taking over a decade in some cases [138]. The timing and risk/benefit profile of candidate interventions therefore need to be carefully considered. Intervening at stage 2, where dysglycemia is evident, permits focus on those at highest risk and decreases the duration of clinical trials; however, it is possible that some treatments may be less effective at this later stage of disease.

There are many other immunotherapy approaches in T1D that are not discussed here due to space considerations. Examples include the non-depleting anti-CD40 antibody Iscalimab (NCT04129528) and the JAK1/JAK2 inhibitor Baricitinib (NCT04774224). There is also interest in repurposing therapies with proven utility in other autoimmune conditions, such as Hydroxychloroquine (NCT03428945) which is used in systemic lupus erythematosus and rheumatoid arthritis, and the IL-12/IL-23 targeting drug Ustekinumab (NCT03941132) which is used in psoriasis.

Antigen-specific immunotherapies are likely to be important contributors to the future therapeutic landscape, and their specificity may prove beneficial from a safety perspective. Early data suggest administering antigen-specific therapies following IL-2-mediated Treg expansion may be a useful strategy [139]. Indeed, there is increasing interest in combination approaches, perhaps leveraging two immunotherapies such as the Rituximab/Abatacept combination currently being tested (NCT03929601), or perhaps combining an immunotherapy with strategies to augment β cell function. The latter goal will be boosted by recent advances in the generation of stem cell-derived β cells [140] and human islet-like organoids [141].

With effective immunotherapies gradually starting to emerge, it will be important to establish mechanisms that allow more individuals to be offered the option of participating in clinical trials at T1D diagnosis so that candidate interventions can be compared. Combined with large-scale initiatives to identify at-risk individuals, such as the trailblazing public health screening approach taken by Ziegler and colleagues [142], it seems that the new landscape for T1D treatment and prevention is beginning to take shape.

## Data Availability

No data are available as this is a review article.

## Abbreviations

ATG: Anti-thymocyte globulin
CD: Cluster of differentiation
CTLA-4: Cytotoxic T lymphocyte-associated antigen 4
Fc: Fragment crystallisable
FMT: Fecal microbiota transplant
GCSF: Granulocyte colony-stimulating factor
HbA1c: Haemoglobin A1c
IFNγ: Interferon gamma
Ig: Immunoglobulin
IL: Interleukin
MMTT: Mixed-meal tolerance test
NK: Natural killer
NOD: Non-obese diabetic
T1D: Type 1 diabetes
TCR: T-cell receptor
Tfh: Follicular helper T cells
TGFβ: Transforming growth factor beta
TIGIT: T-cell immunoreceptor with Ig and ITIM domains
TNFα: Tumour necrosis factor-alpha
TNFR: Tumour necrosis factor receptor
Treg: Regulatory T cell
ZnT8: Zinc transporter 8
